# Visual Field Examinations for Retinal Diseases: A Narrative Review

**DOI:** 10.3390/jcm14155266

**Published:** 2025-07-25

**Authors:** Ko Eun Kim, Seong Joon Ahn

**Affiliations:** 1 Department of Ophthalmology, Asan Medical Center, Ulsan University College of Medicine, Seoul 05505, Republic of Korea; csckek@gmail.com; 2 Department of Ophthalmology, Hanyang University Hospital, Hanyang University College of Medicine, Seoul 04763, Republic of Korea

**Keywords:** visual fields, retinal disease, screening, progression

## Abstract

Visual field (VF) testing remains a cornerstone in assessing retinal function by measuring how well different parts of the retina detect light. It is essential for early detection, monitoring, and management of many retinal diseases. By mapping retinal sensitivity, VF exams can reveal functional loss before structural changes become visible. This review summarizes how VF testing is applied across key conditions: hydroxychloroquine (HCQ) retinopathy, age-related macular degeneration (AMD), diabetic retinopathy (DR) and macular edema (DME), and inherited disorders including inherited dystrophies such as retinitis pigmentosa (RP). Traditional methods like the Goldmann kinetic perimetry and simple tools such as the Amsler grid help identify large or central VF defects. Automated perimetry (e.g., Humphrey Field Analyzer) provides detailed, quantitative data critical for detecting subtle paracentral scotomas in HCQ retinopathy and central vision loss in AMD. Frequency-doubling technology (FDT) reveals early neural deficits in DR before blood vessel changes appear. Microperimetry offers precise, localized sensitivity maps for macular diseases. Despite its value, VF testing faces challenges including patient fatigue, variability in responses, and interpretation of unreliable results. Recent advances in artificial intelligence, virtual reality perimetry, and home-based perimetry systems are improving test accuracy, accessibility, and patient engagement. Integrating VF exams with these emerging technologies promises more personalized care, earlier intervention, and better long-term outcomes for patients with retinal disease.

## 1. Introduction

The visual field (VF) represents the entire area an individual can perceive when fixating on a central point, encompassing both central and peripheral vision [1]. It is often conceptualized as an “island of vision surrounded by a sea of blindness,” with the fovea—the central part of the retina responsible for the sharpest vision—representing the peak of sensitivity. VF testing, or perimetry, is a psychophysical method designed to systematically measure the sensitivity of the retina to light across this field [1,2]. Its primary objectives include detecting localized areas of vision loss (scotomas), quantifying the extent and depth of visual impairment, and monitoring changes over time [1,2,3].

Beyond its well-established role in glaucoma, perimetry is a critical functional assessment tool for a diverse range of retinal diseases and other ocular and neurological conditions [1]. It provides crucial functional information that complements structural imaging modalities—such as optical coherence tomography (OCT) and fundus photography—offering insights into how various pathologies impact visual perception [1,4]. Because there is a direct topographical correspondence between a VF defect and the retinal site of damage, changes in the retina directly translate to measurable functional deficits, often even before structural changes are clinically obvious [5,6]. This highlights VF testing’s role as a functional indicator for early disease detection and progression, providing a proactive tool in retinal disease management [6].

The journey of perimetry began with qualitative, rudimentary methods such as the confrontation VF test [2]. A significant leap occurred with the introduction of the manual bowl projection perimeter—most notably, the Goldmann perimeter—which standardized testing parameters such as test distance, background illumination, stimulus size, and intensity, facilitating comprehensive central and peripheral field testing [2]. The advent of automated perimetry—exemplified by the Humphrey Field Analyzer (HFA, introduced in 1981)—marked a pivotal shift, enhancing sensitivity, reproducibility, and quantitative analysis, and enabling more precise detection and monitoring of VF loss over time [2,7,8]. This historical progression—from qualitative manual methods to quantitative automated perimetry and now to advanced imaging-integrated techniques, such as microperimetry—reflects a continuous drive toward greater precision, objectivity, and earlier detection in ophthalmology [1]. Early and accurate detection of retinal pathologies is critical for preventing irreversible vision loss and minimizing disease progression, as evidenced by drug-induced retinopathies, where early detection can halt serious progression [9,10].

This paper represents a narrative review of perimetric methods and their applications in retinal diseases. Key articles were identified through targeted searches in PubMed and Embase, supplemented by manual review of reference lists from major reviews. This review includes the specific techniques employed, the findings of VF defects observed in retinal diseases, and the clinical utility of these examinations in diagnosis, monitoring, and prognosis. Key retinal pathologies to be discussed include age-related macular degeneration (AMD), diabetic retinopathy (DR) and diabetic macular edema (DME), drug-induced retinopathies, and inherited retinal dystrophies such as retinitis pigmentosa (RP). Furthermore, the paper addresses the inherent challenges in VF testing and explores the promising landscape of emerging technologies and future directions in this field.

## 2. Principles and Methodologies of VF Testing

### 2.1. Basic Concepts of Perimetry

The VF is commonly conceptualized as a “hill of vision”, a three-dimensional representation of retinal sensitivity [11]. The fovea forms the apex of this “hill”, exhibiting the highest sensitivity to light. As one moves toward the periphery, sensitivity gradually declines. Localized areas of diminished or absent vision are termed “scotomas [12]”. Scotomas can be classified as relative—where only dim stimuli fail to be perceived but brighter or larger stimuli remain visible—or absolute—where no light stimulus can be detected within the affected area. A normal physiological blind spot corresponds to the optic disk, which lacks photoreceptors; this natural absolute scotoma is typically located 10–20° temporal to the point of fixation [12].

In automated perimetry, retinal sensitivity to light is quantitatively measured in decibels (dB). This logarithmic scale inversely relates to the intensity of the light stimulus, which is measured in apostilbs (asb). A higher dB value signifies greater retinal sensitivity—indicating the ability to detect dimmer lights—whereas 0 dB represents the brightest stimulus (lowest sensitivity) [13]. Failure to detect a 0 dB stimulus denotes an absolute scotoma. This quantitative measurement system translates subjective patient perception into standardized data, enabling objective assessment of retinal function, comparison over time, across patients, and against age-matched normative databases [8].

### 2.2. Traditional Perimetry Techniques

Traditional VF testing encompasses several techniques (Table 1), ranging from simple qualitative screenings to more sophisticated automated methods.

#### 2.2.1. Confrontation VF Test

Confrontation VF test is a rapid, qualitative screening method performed in a face-to-face setting. The examiner moves fingers or a target into the patient’s peripheral vision, and the patient indicates when the object becomes visible [1]. While quick to administer, this method is highly subjective and lacks the sensitivity required for detecting subtle or early defects, particularly in asymptomatic patients. Its utility is primarily for gross screening rather than detailed diagnostic assessment [1].

#### 2.2.2. Kinetic Perimetry (e.g., Goldmann Perimetry)

In kinetic perimetry, a stimulus of fixed size and brightness is moved from a non-seeing area into the patient’s VF. The patient signals the moment the stimulus becomes visible, and these points are recorded to plot isopters—lines connecting points of equal differential light sensitivity [1,14]. The Goldmann perimeter (first commercialized in the 1940s) standardized this process, allowing comprehensive testing of both central and peripheral fields [2]. Because objects in daily life often enter the VF via movement, kinetic perimetry remains relevant. In RP, for example, Goldmann kinetic perimetry is still the clinical standard for mapping and monitoring progressive peripheral-to-central vision loss [15,16].

#### 2.2.3. Automated Static Perimetry

Automated static perimetry presents stationary, dim light stimuli of varying intensities at fixed points within a bowl-shaped perimeter. The patient responds by pressing a button when a light is perceived; the machine automatically determines the threshold (the dimmest light seen 50% of the time) for each tested location, thereby generating a detailed, quantitative map of retinal sensitivity [17].

**Figure 1:**
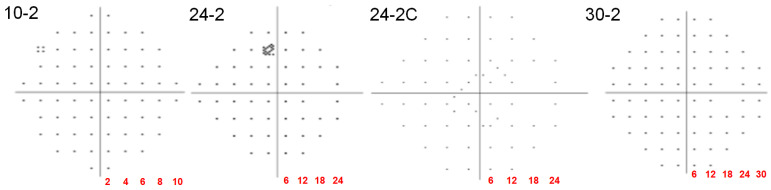
Test grid patterns for commonly used Humphrey visual field protocols: 10-2, 24-2, 24-2C, and 30-2.

The HFA offers several specialized protocols (Figure 1) [6,14]:

10-2 protocol measures the central 10° of the VF (68 points). Crucial for evaluating optic disk, including advanced glaucoma, and retinal, particularly macular conditions. In hydroxychloroquine (HCQ) retinopathy, the 10-2 HVF is a key screening test due to its high sensitivity in detecting early parafoveal toxicity [18]. 24-2 protocol assesses 24° temporally and 30° nasally (54 points). This is commonly used for general glaucoma screening, early detection, and neuro-ophthalmic conditions. 30-2 measures 30° temporally and nasally (76 points) and is also employed for general screening and neurological conditions. In cases of localized macular or extramacular changes (e.g., HCQ retinopathy), expanded grids (such as 24-2C, 30-2, and 60-4) or custom point placement within the 2–6° parafoveal ring might enhance sensitivity to detect early scotomas.

24-2C protocol integrates the 24-2 grid with 10 additional points in the macular area [19,20], providing a more comprehensive assessment of both central and wider fields (Figure 1). Comparative studies have demonstrated that the 24-2C VF test, which incorporates 10 additional central test points, shows moderate-to-substantial agreement with the 10-2 test in detecting central VF defects (κ = 0.55–0.65), with both tests exhibiting similar sensitivity and specificity [21]; however, the 10-2 test may detect approximately 10% more central defects than 24-2C, indicating that 24-2C offers a practical balance between comprehensive central field assessment and reduced testing time. However, the utility of the 24-2C protocol in retinal diseases such as diabetic retinopathy or age-related macular degeneration remains underexplored, with a lack of direct comparative studies, thereby warranting future research to establish its role in these conditions.

HFA uses algorithms such as the Swedish Interactive Thresholding Algorithm (SITA) (Standard, Fast, Faster) to optimize testing efficiency and accuracy [22]. SITA dynamically adjusts stimulus presentation based on patient responses, significantly reducing test duration while maintaining reliability [22,23]. Results are compared against age-matched normative databases to identify significant vision loss [24]. Automated static perimetry—especially with efficient algorithms like SITA—revolutionized VF testing by enabling standardized, quantitative, and expedited assessments, markedly improving the detection of subtle and early changes in conditions like HCQ retinopathy [18,25].

The value of serial automated static perimetry for tracking disease progression has been demonstrated in large, prospective trials. In the Early Manifest Glaucoma Trial (EMGT), newly diagnosed glaucoma patients underwent serial Humphrey testing for up to six years, defining rates of field loss [26]. Similarly, the Ocular Hypertension Treatment Study (OHTS) showed that treatment delays progression of VF loss in ocular hypertension [27], and the Collaborative Initial Glaucoma Treatment Study (CIGTS) compared long-term outcomes between medical and surgical therapy [28].

#### 2.2.4. Frequency Doubling Technology (FDT)

FDT perimetry leverages a flicker illusion created by a counterphase flickering of a low-spatial-frequency sinusoidal grating at a high temporal frequency, making the image appear to have double its actual spatial frequency. This is hypothesized to preferentially target the magnocellular (M) retinal ganglion cell pathway, enabling earlier detection of nerve fiber layer dysfunction [29,30]. Initially developed as a rapid screening tool for early glaucoma—because of its sensitivity to retinal ganglion cell loss—FDT has also demonstrated utility in detecting early neural changes in DR, even before clinically visible microvascular changes appear [31]. FDT’s capacity to isolate specific retinal ganglion cell pathways offers mechanistically targeted insights, potentially allowing earlier intervention with neuroprotective strategies before widespread damage occurs [30].

### 2.3. Advanced and Specialized Perimetry Techniques

#### 2.3.1. Microperimetry

Retinal microperimetry (MP) assesses retinal sensitivity while providing real-time, direct observation of the fundus [32]. An eye-tracking system compensates for involuntary eye movements during testing, ensuring that sensitivity measurements are precisely correlated with specific anatomical locations on the retina [32]. Stimulus sizes are comparable to standard automated perimetry and cover up to 30° from the fovea.

Microperimetry overcomes limitations of conventional perimetry—particularly insensitivity to small scotomas and inability to precisely identify fixation characteristics. It is highly valuable for macular diseases (AMD, DME, Stargardt disease), detecting functional impairments before structural abnormalities are visible on retinal imaging [33,34,35]. MP is also crucial for assessing treatment efficacy, such as anti-vascular endothelial growth factor (anti-VEGF) injections in AMD or DME, by repeatedly testing identical macular sites over time [34,36]. Furthermore, microperimetry is beneficial in presurgical and postsurgical evaluations. By providing pixel-level functional mapping and correlating it with anatomical landmarks, MP grants a more granular view of functional deficits, which is vital for understanding focal retinal pathologies and guiding management [33,34,35].

#### 2.3.2. Amsler Grid

The Amsler grid is a simple square grid pattern with a central fixation dot, typically printed on paper for patient self-monitoring of visual distortions (metamorphopsia) or central blind spots (scotomas) [37]. The patient covers one eye, focuses on the central dot, and notes any areas where lines appear wavy, blurry, dark, or blank [38,39].

It is considered a gold standard for detecting metamorphopsia, especially in macular diseases like wet AMD and DME. In dry AMD, Amsler grid use can prompt timely detection of conversion to the wet form, enabling early intervention [40,41]. However, it is not as reliable for detecting subtle decreases in retinal sensitivity in the earliest, asymptomatic stages of conditions like HCQ retinopathy [25]. The widespread use of the Amsler grid for AMD exemplifies a successful patient self-monitoring model for a specific symptom (metamorphopsia) [41], but its subjective nature and limited sensitivity underscore its role as a screening tool rather than a definitive diagnostic instrument.

## 3. VF Findings in Retinal Diseases

We conducted targeted searches of PubMed and Embase for English-language articles published between January 2000 and June 2025. Search terms combined (“visual field” OR perimetry OR microperimetry OR “frequency-doubling perimetry”) AND (“retinal disease” OR “macular disease” OR “hydroxychloroquine” OR “pentosan polysulfate” OR “age-related macular degeneration” OR “diabetic retinopathy” OR “inherited retinal disease” OR retinitis pigmentosa). We prioritized original human studies, review articles, and consensus guidelines in peer reviewed journals, excluding case reports with fewer than three patients, non–peer reviewed abstracts, and non-English reports. In addition, we included a small number of classic, foundational studies published before 2000 to provide historical context for the evolution of perimetric methods. Reference lists of key articles were hand searched to capture any further relevant literature. While we did not formally grade evidence in this narrative review, we recognize the varying strength of included studies. Randomized controlled trials (RCTs), where available, provide the most robust evidence for perimetric performance, followed by prospective observational studies and retrospective case series. Most perimetry studies in retinal diseases fall into lower tiers of this hierarchy, which limits generalizability and warrants cautious interpretation.

VF defects manifest in distinct patterns depending on the underlying retinal pathology, providing crucial diagnostic and prognostic information (Table 2).

### 3.1. AMD

AMD is the leading cause of severe central vision loss in individuals over 60 [42]. It primarily affects the macula, the central retina responsible for detailed vision, reading, and facial recognition. While AMD rarely causes complete blindness, it significantly impairs central visual function [42,43].

VF defects in AMD are predominantly central. Patients often report blurry or fuzzy vision, difficulty recognizing faces, and a dark or empty area (central scotoma) in their VF [44]. In wet AMD, straight lines appear crooked or wavy (metamorphopsia) due to choroidal neovascular membranes leaking fluid and blood under the macula, potentially leading to a large central blind spot [45]. The Amsler grid is widely used for patient self-monitoring of these distortions, facilitating early detection of conversion from dry to wet AMD and prompting timely treatment [40].

In geographic atrophy (GA)—the advanced stage of dry AMD—patients experience gradual central vision loss or dark, blurry areas in the center of vision [46]. GA often begins in the region adjacent to the fovea (non-central GA) and progresses into the fovea, resulting in severe central vision loss over time. The presence of drusen—tiny yellow deposits under the retina—is an early sign of AMD and can be associated with subtle paracentral scotomas on microperimetry before overt VF defects appear on standard perimetry [47].

Few underpowered studies directly compare microperimetry to conventional perimetry in AMD. Furthermore, perimetry may be less sensitive than structural imaging, particularly in early or dry forms of the disease where subtle changes are better captured by OCT or fundus autofluorescence.

### 3.2. DR and DME

DR is a common microvascular complication of diabetes, directly correlated with long-term glycemic control [48]. Damage to small retinal blood vessels leads to fluid and blood leakage, causing retinal swelling and blurred vision. DME involves fluid accumulation in the macula and is the most frequent cause of vision loss in both proliferative and nonproliferative DR [49]. Symptoms include seeing spots or floaters, blurred vision, difficulty seeing at night, and a dark or empty spot in central vision. Metamorphopsia and central scotoma are common in DME due to disruption of normal retinal architecture [42].

VF loss—particularly detected by FDT perimetry—is an early sign of diabetic neuroretinopathy, often preceding clinically detectable microvascular DR [50,51]. Patients with diabetes but no visible DR are more likely than non-diabetic individuals to have visual subfield defects [31]. These defects become more frequent and severe with the onset and progression of clinically apparent retinopathy. In early DR, VF defects tend to be diffuse; in later stages, they occur preferentially in the nasal fields and often symmetrically. This pattern suggests inner retinal sensory neuropathy associated with diabetes itself—independent of microvascular changes. However, blue-on-yellow (B/Y) perimetry has proven more sensitive than conventional methods for detecting VF defects [52].

Early VF loss by FDT can precede clinical retinopathy, but these findings often lack external validation. In early disease, imaging often detects structural changes before perimetric loss becomes apparent. Sensitivity and specificity of various perimetric methods vary widely, and direct, large-scale comparative studies are lacking. Microperimetry can localize functional deficits in DME, but large validation studies comparing it to standard perimetry are absent. Regulatory acceptance and logistical barriers such as the need for dedicated training and longer test durations remain significant issues.

### 3.3. Drug-Induced Retinopathies

HCQ retinopathy poses a significant risk for irreversible visual loss, which can progress even after drug cessation [18]. Early detection is paramount to minimize progression and preserve vision. The prevalence of HCQ retinopathy is higher than previously estimated—affecting approximately 7.5% of patients taking the drug for over five years and rising to nearly 20% after 20 years of treatment [53].

**Figure 2:**
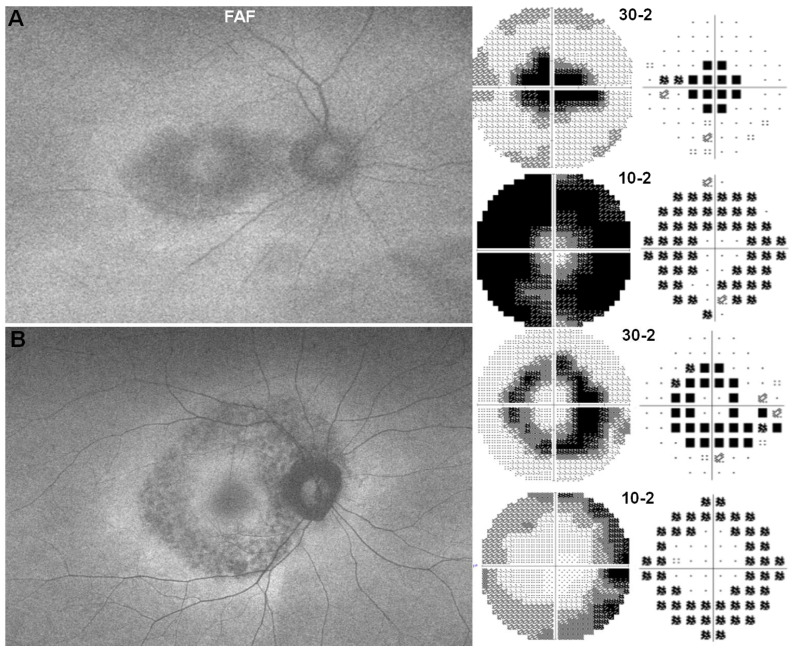
Representative fundus autofluorescence images and corresponding visual field defects in (**A**) parafoveal and (**B**) pericentral patterns of retinopathy.

VF testing, particularly the 10-2 protocol, is a key component of HCQ retinopathy screening [18] (Figure 2). Early HCQ retinopathy often presents with subtle, nonspecific paracentral defects that may require repeated testing for confirmation [10]. The classic pattern is a complete or incomplete ring scotoma, typically located between 2° and 6° from fixation, with relative sparing of the fovea [10,18]. In some populations—particularly Asian patients—an extramacular or pericentral pattern of damage may occur, involving retinal changes 8° or more from fixation, underscoring the importance of wider screening tests (e.g., 24-2 or 30-2 HVF) for these groups [10,18] (Figure 2B).

Pentosan polysulfate sodium (PPS), prescribed for interstitial cystitis, has recently been implicated in a distinct maculopathy characterized by fundus pigmentary changes and corresponding VF defects [54]. Long-term PPS use (cumulative dose > 500 g and duration ≥ 10 years) has been associated with paracentral scotomas, often appearing as bilateral, symmetric relative depressions on 10-2 HVF testing [54,55]. These scotomas may correlate topographically with areas of macular pigment mottling and drusen-like deposits on OCT and fundus autofluorescence [54]. Similar to HCQ retinopathy, PPS-associated defects can progress despite drug discontinuation, highlighting the importance of incorporating periodic 10-2 screening—especially in patients with long-term or high-dose PPS exposure—to detect early functional changes and guide clinical management.

Tamoxifen, used in breast cancer therapy, can induce crystalline deposits in the macula and peripheral retina, occasionally causing foveal cone loss with corresponding scotomas detectable on 10-2 testing [56]. Deferoxamine, an iron-chelation agent, can cause pigmentary retinopathy and RPE atrophy, resulting in ring-shaped or paracentral defects on 24-2 or 30-2 testing, which can be monitored using automated static perimetry.

While perimetry plays a key role in screening for toxic retinopathies, its sensitivity may vary by disease stage and pattern, and in some cases, imaging modalities such as OCT or autofluorescence may detect early structural changes before functional deficits are evident. Although 10-2 protocol is the standard for HCQ toxicity screening, comparisons with newer modalities are limited by small sample sizes and inconsistent definitions of toxicity. For PPS maculopathy, most data come from retrospective series, with no prospective trials or regulatory guidance for VF monitoring. The lack of standardized criteria complicates early detection and study comparisons.

### 3.4. Retinitis Pigmentosa (RP)

Retinitis pigmentosa (RP) comprises a group of inherited retinal degenerations characterized by progressive loss of rod and cone photoreceptors and RPE dysfunction [57]. Clinically, patients experience night blindness (nyctalopia), photophobia, and progressive constriction of VFs [57].

The classical progression in RP involves patchy loss of peripheral VFs that coalesce into a ring scotoma, eventually leading to “tunnel vision” and, in advanced stages, legal blindness [15,58]. Night vision impairment is usually the first symptom, as rods degenerate before cones. As the disease advances, patients lose midperipheral vision followed by far peripheral fields, often preserving central vision until the final stages [15,58]. The ring scotoma typically begins as isolated scotomas approximately 20° from fixation, which gradually merge into a partial, then complete, ring [15]. Over time, the outer edge of this ring moves peripherally faster than the inner edge constricts centrally.

Goldmann perimetry remains the preferred method for tracking RP’s peripheral field loss and ring scotoma evolution [16]. Studies show that fields deteriorate more rapidly when testing smaller stimuli [15,16]. VF testing is invaluable for monitoring disease progression in RP and for documenting patients’ status regarding legal blindness for driving and disability certification [15,16,58].

However, the subjectivity and operator dependence of Goldmann kinetic perimetry are recognized limitations. Comparative studies with automated perimetry are small, and no large trials have established automated perimetry as regulatory endpoints. Although VF testing is valuable in retinitis pigmentosa, imaging biomarkers such as hypoautofluorescent areas on fundus autofluorescence and ellipsoid zone width or retinal thickness measurements on OCT can detect disease progression earlier and more objectively.

### 3.5. Inherited Macular Dystrophy

Stargardt disease (juvenile macular dystrophy) is an autosomal recessive disorder caused by mutations in the ABCA4 gene, leading to lipofuscin accumulation in the RPE and progressive macular atrophy [59,60]. Patients typically experience bilateral central vision loss in the first or second decade of life [61]. When tested with automated static perimetry, central relative or absolute scotomas are common, reflecting photoreceptor and RPE loss in the macula [62]. The pattern of retinal sensitivity loss correlates closely with autofluorescence imaging [63]; however, perimetry may underestimate disease severity due to limited sensitivity compared to OCT and fundus autofluorescence especially at early stages.

Inherited macular dystrophies such as Best disease and cone dystrophies produce characteristic central and paracentral scotomas that warrant tailored perimetric strategies. Best disease typically presents in childhood with subretinal “egg-yolk” lesions and later atrophy. Automated 10-2 perimetry reveals central absolute or relative scotomas corresponding to lesion location. Microperimetry may better capture changes in fixation and preferred retinal loci. Cone/cone–rod dystrophies typically manifest with early central scotomas [64]. These are reliably detected by 10-2 automated static perimetry and microperimetry, providing valuable functional correlation with structural loss. However, fixation instability can affect test reliability, which should be mitigated by using eye-tracking perimeters and close monitoring of fixation stability during testing.

Automated static perimetry correlates with fundus autofluorescence–defined lesions, but large natural history studies using perimetry are lacking. Microperimetry may detect sensitivity loss earlier, but findings are from small, uncontrolled studies. Automated perimetry is seldom used as a regulatory endpoint in clinical trials, as fixation variability complicates comparisons.

### 3.6. Central Serous Chorioretinopathy (CSCR)

Central serous chorioretinopathy (CSCR) is characterized by the serous detachment of neurosensory retina at the macula, often secondary to choroidal hyperpermeability [65]. Patients typically present with blurred or distorted central vision, micropsia, and metamorphopsia [65,66]. VF testing generally reveals a relative central scotoma or paracentral relative depression corresponding to the area of serous detachment. The size and depth of the scotoma often correlate with OCT findings of subretinal fluid height [67]. Because serous detachments can fluctuate, serial perimetry may demonstrate changes in central sensitivity that parallel fluid resolution with treatment or spontaneous remission [68]. In addition to perimetry, the Amsler grid offers a rapid and patient-driven means to detect and monitor central metamorphopsia and scotomas in CSCR, particularly useful during periods of fluctuating serous detachment.

However, most perimetric data in CSCR come from small, retrospective series with methodological heterogeneity. Although automated perimetry reflects the functional impact of serous detachment, OCT remains more sensitive for detecting subtle fluid changes and is typically prioritized for diagnosis and monitoring. Practical barriers, such as test duration and the sufficiency of OCT, limit routine field testing.

### 3.7. Retinal Vein Occlusion (RVO)

Retinal vein occlusion (branch or central) produces ischemic and edematous changes in the inner retina, leading to VF defects that correspond to the area of venous drainage obstruction [69]. In branch RVO (BRVO), patients often exhibit sectoral scotomas—congruent, arcuate, or altitudinal defects—corresponding to the quadrant of occlusion [69]. Central RVO (CRVO) can cause diffuse depression of sensitivity, greater central loss, and peripheral field defect [70]. Perimetry findings correlate with the extent of capillary nonperfusion on fluorescein angiography—areas of nonperfusion correspond to absolute scotomas or deep depressions on static perimetry. Serial perimetry can be useful for monitoring treatment response (e.g., anti-VEGF therapy) by demonstrating improvement in sensitivity as macular edema resolves. Additional insights can be gained from microperimetry and OCT angiography, which detects focal perfusion deficits in the macula [71].

However, correlations between sectoral scotomas and angiographic findings are based on small cohorts, and thresholds for meaningful change are not well defined. Improvement in sensitivity after therapy is only reported in small series. While perimetry provides functional correlates of perfusion loss, angiographic imaging modalities such as fluorescein angiography and OCT angiography more sensitively detect ischemic zones, particularly areas of capillary nonperfusion, in eyes with RVO.

### 3.8. Macular Hole

Full-thickness macular hole (FTMH) is characterized by a defect in the foveal retina, often secondary to vitreomacular traction [72]. Patients typically present with decreased central visual acuity, metamorphopsia, and a central scotoma [73,74]. Automated 10-2 perimetry reveals an absolute central scotoma corresponding to the anatomic hole; the size of this scotoma often approximates the OCT-measured hole diameter. After surgical repair (pars plana vitrectomy with ILM peeling and gas tamponade), perimetry can document recovery of central sensitivity in cases of successful hole closure [75]; residual or irregular scotomas may persist and correspond to areas of photoreceptor disruption seen on postoperative OCT. Microperimetry is particularly sensitive for postoperative improvement of retinal sensitivity [75]. Moreover, the simplicity and accessibility of the Amsler grid make it an effective tool for patients to self-monitor central distortion and scotoma size both pre- and post-operatively, complementing clinical OCT and perimetric assessments.

However, most data on post-surgical perimetry come from small case series. Microperimetry offers high-resolution mapping, but its added prognostic value over standard testing is unproven, and lack of standardized criteria limits broader adoption. In contrast, OCT provides structural detail and prognostic information, particularly in assessing surgical outcomes in eyes with FTMH, leading to its widespread use in this setting.

### 3.9. Miscellaneous Retinal Diseases

Patients with macular telangiectasia type 2 often develop paracentral scotomas corresponding to telangiectatic and atrophic changes around the fovea. Microperimetry can precisely map these localized sensitivity deficits, whereas standard 10-2 perimetry may underestimate the true extent of functional loss [76]. Autoimmune retinopathy frequently presents with diffuse or ring-like VF defects that can precede ophthalmoscopic findings. Serial 24-2 automated static perimetry may be useful for tracking progression and guiding immunosuppressive management in conjunction with structural findings (e.g., OCT) and electroretinography.

## 4. Clinical Utility of VF Examinations in Retinal Diseases

VF testing serves multiple critical functions in the management of retinal diseases, extending from initial diagnosis to long-term monitoring and prognosis. In clinical practice, patients with early AMD or other retinal diseases often start with Amsler grid screening, and those reporting distortions or defects may proceed to structural imaging and/or perimetry—selecting the specific test based on disease characteristics, local availability, and patient factors (e.g., fixation stability).

### 4.1. Diagnosis

VF tests are essential for detecting blind spots and mapping their size, shape, and location, thereby helping to identify how specific pathologies affect vision. In drug-induced retinopathies, VF changes often precede OCT abnormalities, making perimetry a vital tool for early, pre-symptomatic detection [77]. The American Academy of Ophthalmology (AAO) guidelines recommend automated VF test as one of primary tests for monitoring patients continuing HCQ therapy [18,77]. Likewise, PPS maculopathy often presents with paracentral scotomas on VF testing that correlate with early pigmentary alterations on fundus autofluorescence, prior to frank macular atrophy [54].

For other macular diseases—such as AMD, CSCR, and Stargardt disease—microperimetry (fundus perimetry) provides high-resolution, topographically precise sensitivity maps that often reveal localized functional deficits at or near the fovea before they are visible on fundus photography or OCT. In early AMD, for example, microperimetry can detect paracentral sensitivity loss corresponding to drusen distribution or early geographic atrophy. In Stargardt disease, early central scotomas on microperimetry align with areas of increased fundus autofluorescence, enabling genotype–phenotype correlations and earlier intervention.

Inherited retinal dystrophies—such as RP and cone–rod dystrophies—often exhibit characteristic VF patterns (e.g., ring scotomas in RP); Goldmann kinetic perimetry remains the gold standard for mapping these peripheral deficits, but automated static perimetry can complement early detection of paracentral sensitivity loss in cone–rod phenotypes. In occult macular dystrophy (OMD), VF testing is also useful for diagnosis, often revealing central scotomas despite a normal fundus appearance [78].

Integrated en face OCT–VF overlays enable precise spatial correlation of photoreceptor and retinal layer abnormalities with corresponding scotomas, facilitating earlier detection and more accurate monitoring. Fusion of en face reflectivity and thickness maps with VF probability plots may improve diagnostic accuracy and guide personalized management decisions by enhancing our understanding of structure–function relationships in retinal diseases.

### 4.2. Monitoring Disease Progression or Recovery

Repeated VF assessments are crucial for tracking stability or progression over time. In progressive retinal dystrophies—such as RP—Goldmann kinetic perimetry quantifies the constriction of peripheral isopters and tracks the diameter of ring scotomas, which directly correlates with functional vision and informs counseling regarding mobility training and driving eligibility [16]. The rate of field constriction can also serve as an outcome measure in gene therapy and stem cell trials.

In drug-induced toxicity, serial HVF testing can detect gradual paracentral sensitivity losses over time [55,79], and documenting this progression aids in stratifying functional risk and determining the need for intervention or low-vision support.

In AMD, VF testing—particularly microperimetry—enables precise monitoring of geographic atrophy expansion by measuring local threshold changes around atrophic borders. After anti-VEGF injections for neovascular AMD, microperimetry can detect functional improvements or stabilization that may not immediately reflect in visual acuity, guiding retreatment intervals. In DME, repeated FDT or Blue-on-Yellow perimetry demonstrates changes in central/paracentral sensitivity that correlate with OCT-measured macular thickness changes; declines in sensitivity can precede OCT-detectable recurrences of edema, prompting earlier intervention.

In RVO, perimetry tracks recovery of function following anti-VEGF or laser therapy. Microperimetry can detect residual macular scotomas that persist after edema resolution, signaling areas where neuroretinal damage occurred and informing visual rehabilitation efforts.

Furthermore, the Amsler grid provides a simple, patient-driven method to self-monitor central metamorphopsia and scotomas in macular conditions such as AMD, DME, CSCR, and FTMH.

### 4.3. Prognosis

VF test results provide prognostic insights by predicting the likely trajectory of visual function. In RP, the annual rate of peripheral isopter constriction or ring scotoma shrinkage correlates with mobility performance and quality-of-life measures, informing discussions about expected functional decline and timing of low-vision aids [16,58]. Moreover, baseline microperimetric sensitivity in sites adjacent to foveal sparing can predict maintenance of reading ability in cone–rod dystrophies and Stargardt disease, guiding vocational counseling.

For HCQ and PPS retinopathies, if early paracentral defects are detected and exposure is discontinued, the likelihood of halting progression and preserving central vision is substantially higher [80]. Thus, perimetric findings directly influence risk stratification and long-term visual prognostication. In AMD, microperimetry threshold values around small drusen or hyperautofluorescent (fundus autofluorescence) spots can predict the rate of progression to geographic atrophy, enabling personalized monitoring intervals and early enrollment in neuroprotective or complement-directed trials. Microperimetry can also be a valuable tool to determine the risk of stratification and progression by monitoring the changes in eyes with AMD [81].

In retinal vascular diseases such as DR and RVO, the extent and depth of initial perimetric defects—particularly when combined with OCT angiography metrics of capillary nonperfusion—correlate with long-term visual outcomes and risk of neovascular complications, guiding early aggressive therapy and frequency of follow-up.

## 5. Challenges and Limitations

Despite its indispensable role, VF testing presents several challenges and limitations, particularly in the context of retinal diseases (Table 3).

### 5.1. Patient Factors

VF testing is a subjective psychophysical procedure, and its reliability hinges on patient cooperation and consistent performance. Beyond fatigue, anxiety, and inattention, additional factors include

Learning Effects: Patients often improve on repeated tests as they become familiar with the procedure. Early sessions may therefore underestimate true sensitivity, whereas subsequent tests may show artificial “improvement” unrelated to disease status.

Cognitive and Language Barriers: Patients with cognitive impairment (e.g., dementia, stroke) or language barriers may misunderstand instructions, yielding unreliable results. Providing instructions in the patient’s primary language and offering demonstration trials can help.

Unstable Fixation: In macular diseases (e.g., AMD, Stargardt, CSCR), central vision loss can lead to eccentric or unstable fixation. Standard automated perimetry assumes stable central fixation; unstable fixation can mislocalize stimuli and produce spurious defects. Microperimetry addresses this by tracking the fundus in real time, but standard perimetry remains susceptible.

Attention and Motivation Fluctuations: Long test durations can cause lapses in attention, especially in patients discouraged by poor vision. Motivation may wane across sessions, increasing missed responses (“false negatives”) and fixation losses.

Physical Limitations: Elderly patients or those with arthritis often struggle to maintain head position or press the response button consistently. Poor dexterity can cause missed responses, and discomfort may lead to early termination or inconsistent effort. Providing chinrest supports, using shorter “Fast” algorithms (SITA-Fast or SITA-Faster), and allowing breaks can improve reliability.

### 5.2. Media Opacities and Comorbidities

Media opacities and other ocular comorbidities can confound interpretation of perimetric results in retinal disease:

Cataracts and Vitreous Opacities: Lens opacification produces a diffuse depression of sensitivity across the field, mimicking diffuse retinal dysfunction. Even early cataracts can cause localized field depression (e.g., posterior subcapsular cataract in the visual axis). Similarly, vitreous floaters or hemorrhage can create transient scotomas in unpredictable locations.

Pupil Size: Small pupils (<2.5 mm) reduce retinal illumination and artificially depress thresholds, particularly in perimeters that do not compensate for pupil size. Pharmacologic dilation or standardized ambient illumination helps ensure consistency.

Refractive Errors: Uncorrected refractive errors—especially high myopia, hyperopia, or astigmatism—cause defocus, leading to threshold elevations and generalized depression. Even a 1 D refractive error can reduce threshold by 1–2 dB; therefore, precise refractive correction at the test distance is essential.

Coexisting Glaucoma: Distinguishing glaucomatous from retinal field defects is challenging when both conditions coexist. Glaucoma often produces nasal steps or arcuate scotomas that respect the horizontal meridian, whereas retinal scotomas often respect the vertical meridian or present as paracentral defects. However, advanced glaucomatous damage can encroach on central fields, and some retinal diseases (e.g., RP) can produce arcuate-like scotomas, blurring the distinction. Correlating perimetric defects with OCT retinal nerve fiber layer (RNFL) and ganglion cell layer maps can help differentiate the two.

Iatrogenic Effects of Retinal Treatments: Panretinal photocoagulation (PRP) for proliferative diabetic retinopathy often induces predictable peripheral scotomas that can result in total field constriction if extensive. Silicone oil tamponade during vitrectomy can cause transient threshold elevations in inferior field locations due to oil meniscus interference. After anti-VEGF injections, transient intraocular pressure spikes can yield temporary field depressions unrelated to disease or its progression.

### 5.3. Normative Database and Floor/Ceiling Effects

Normative Database Mismatch: Automated perimeters rely on age-matched normative databases. Standard normative databases may not suit all populations; groups with demographic or clinical characteristics that differ from the reference cohort warrant particular consideration. Many databases exclude patients with high myopia (>6 D), high refractive error, or coexisting ocular pathology. Applying these normative values to atypical populations can misclassify normal variations as pathologic or vice versa. This is especially pertinent in inherited dystrophies—where baseline fields differ markedly from the “normal” eye—and in populations with racial/ethnic differences not represented in standard databases.

Floor Effects: In advanced retinal disease (e.g., end-stage RP, advanced geographic atrophy), many test locations may register 0 dB (floor). Once sensitivity reaches 0 dB, further progression cannot be quantified. Patients can appear “stable” on perimetry even as disease worsens. Employing suprathreshold kinetic perimetry or microperimetry (which can detect residual sensitivity below 0 dB thresholds of standard perimeters) helps overcome this limitation.

Ceiling Effects: In early or mild field loss, threshold values may cluster near the upper limit of device sensitivity (e.g., 35–40 dB). Small improvements or declines (e.g., 2 dB) at high-sensitivity locations may fall within test–retest variability, making early change detection unreliable. Advanced AI-driven thresholding algorithms can dynamically modulate stimulus intensity, effectively extending the measurable range and minimizing floor and ceiling effects.

### 5.4. Technical and Environmental Factors

Technical and environmental factors can introduce systematic biases in perimetric measurements, compromising the accuracy and comparability of VF results.

Test Environment: Ambient lighting must be controlled; stray light can elevate background luminance inside the bowl, raising thresholds. Inadequate machine calibration (e.g., bulb aging, incorrect bowl reflectance) introduces systematic bias. Regular calibration and maintenance are required to ensure consistent stimulus luminance and background levels.

Stimulus Presentation Algorithms: Different testing algorithms (e.g., SITA-Standard vs. SITA-Fast vs. SITA-Faster) trade off speed for accuracy. While SITA-Fast and SITA-Faster reduce test time—beneficial for fatigued or elderly patients—they increase variability in threshold estimates, potentially missing subtle defects or changes over time.

Stimulus Size and Location Density: Standard 24-2 and 30-2 grids test at relatively coarse intervals (6° spacing), potentially missing small, localized scotomas (e.g., in early HCQ retinopathy or small geographic atrophy). Microperimetry or 10-2 grids (2° spacing) improve spatial resolution but at the cost of longer testing times and greater patient burden.

Machine Variability Across Devices: Differences between perimeters (e.g., Humphrey vs. Octopus) in stimulus generation, background luminance, and normative databases can make direct comparison of results problematic. Inter-device variability can confound multicenter trials or follow-up when patients switch clinics. Device settings should be standardized across clinics to improve comparability between studies using different device configurations.

### 5.5. Disease-Specific Limitations

In HCQ retinopathy, paracentral scotomas may be missed on 24-2 testing if localized, early damage is positioned within the central zone. Expanded grid patterns (e.g., 24-2C, 30-2) or customized testing locations may be necessary to fully characterize pericentral and extramacular defects as 10-2 can miss the detection.

In Stargardt disease, progressive central atrophy can lead to eccentric fixation, rendering standard center-fixation perimetry unreliable. Microperimetry is essential for patients who adopt stable preferred retinal loci, but many clinics lack this capability.

Accordingly, clinicians should consider expanded grid patterns tailored to at risk regions and integrate multimodal imaging data (such as OCT and fundus autofluorescence) to improve detection and structure function correlation of retinal diseases. While several perimetry devices have longstanding FDA clearance and CE marking, no dedicated AI-based VF analysis software has received regulatory approval.

## 6. Emerging Technologies and Future Directions

The field of ophthalmology is undergoing a profound transformation, driven by novel therapeutic innovations, advanced diagnostic technologies, and integration of artificial intelligence (AI), machine learning (ML), and virtual reality (VR). These advancements hold immense promise for enhancing VF examinations in retinal diseases.

### 6.1. AI and Machine Learning in VF Analysis

AI and ML are revolutionizing ophthalmology by improving disease detection and treatment planning. AI-powered deep learning models—leveraging predictive analytics and retinal image analysis—have achieved high accuracy in identifying eye conditions [82,83]. For example, AI-based programs can analyze VF analysis in seconds, facilitating faster and more accurate diagnoses and identifying subtle patterns that might be missed by human graders. Li et al. trained a convolutional neural network on probability deviation maps from 3712 training and 300 validation VFs, achieving an AUC of 0.96 with 93.2% sensitivity and 82.6% specificity for detecting glaucomatous field loss [84]. Although specific AI applications in VF testing are still emerging, algorithms have been developed to detect preperimetric glaucoma with high precision [85]. AI can also predict VF progression by analyzing large datasets of longitudinal perimetry results, streamlining disease monitoring and risk stratification. Additionally, future perimetry systems should integrate adaptive AI-driven testing that refines point density in regions of suspected damage and employ enhanced sensitivity algorithms to overcome floor and ceiling limitations, thereby improving both the speed and accuracy of detecting early, localized field loss. Furthermore, AI-driven perimetry can minimize test–retest variability and fatigue by using adaptive threshold algorithms to focus on areas of change, thus shortening test duration, and by monitoring reliability metrics in real time to trigger brief pauses or recalibration when needed.

Although various perimetry devices have long held FDA clearance and CE marking, and AI powered OCT platforms such as Altris IMS secured FDA 510 (k) clearance in 2023, no AI based software specifically for perimetry analysis has yet achieved regulatory approval, representing an additional hurdle.

### 6.2. Virtual Reality (VR) Perimetry

Standard automated perimetry remains the gold standard but can be costly and requires a dedicated testing environment, which may be inconvenient for patients with mobility or access issues. VR-based perimetric devices have emerged as promising alternatives for measuring VFs [86]. VR headsets provide an immersive and controlled testing environment, minimizing distractions and ambient light interference, which can improve test reliability and patient engagement. McLaughlin et al. demonstrated good test–retest reliability and strong agreement with standard automated perimetry in both healthy subjects and those with stable field defects [87]. VR perimetry has the potential to revolutionize eye care by improving accessibility in underserved or remote areas, reducing equipment costs, and increasing patient comfort. A systematic review of 64 studies (36 devices) found that VR-headset perimeters perform comparably to gold-standard perimetry, with added benefits in cost, portability, patient comfort, and even built-in rehabilitation modules [88]. Beyond diagnostics, VR is also being explored for vision rehabilitation—using AI-driven eye-tracking to develop targeted rehabilitation programs for patients with field defects [89].

### 6.3. Home-Based Perimetry

The burden of frequent in-office monitoring—especially for retinal diseases requiring anti-VEGF injections—has spurred the development of home-based monitoring strategies. Traditional methods like the Amsler grid offer low sensitivity for early changes, but newer innovations are emerging. More recently, home OCT devices have demonstrated feasibility in preliminary studies [90,91]. For example, Blinder et al. performed a home OCT feasibility study in newly diagnosed nAMD patients, achieving successful self-imaging with high quality—86.5% of 2304 scans met eligibility criteria [90]. These home-based technologies have the potential to mitigate barriers to care—such as travel and scheduling burdens—while enabling timely treatment tailored to individualized disease activity changes. A recent systematic review of 12 studies (3539 participants) and found that home/remote OCT yields diagnostic-grade images, correlates excellently with clinic OCT, and enjoys high patient acceptability across AMD, glaucoma, and diabetic retinopathy [92].

### 6.4. Implementation Barriers for Emerging Technologies

Despite these technological advances, several practical challenges must be addressed before widespread deployment. High initial costs and uncertain reimbursement policies for AI enabled imaging and home perimetry devices may limit adoption in resource constrained settings. Clinician and patient training requirements are substantial, as effective use of these systems depends on standardized acquisition protocols and software proficiency. Data security, privacy concerns, and interoperability with existing electronic health record systems further complicate integration into routine practice. Finally, real world cost effectiveness studies and clear regulatory pathways will be essential to facilitate payer coverage and clinician acceptance.

## 7. Conclusions

VF examinations remain a cornerstone in the diagnosis, monitoring, and prognostic assessment of a wide array of retinal diseases. The evolution from rudimentary confrontation tests to sophisticated automated perimetry such as the HFA with specialized protocols has significantly enhanced the precision and sensitivity of detecting functional deficits. FDT further refined early detection by targeting specific retinal ganglion cell pathways, making it invaluable in conditions like DR. Advanced techniques such as microperimetry represent a substantial leap forward by providing real-time, anatomically correlated functional mapping—crucial for managing focal macular pathologies and assessing treatment responses. Simple tools like the Amsler grid empower patients for self-monitoring of metamorphopsia in AMD.

Despite inherent challenges/limitations, the continuous refinement of VF testing methodologies reinforces its critical role in retinal disease management. The integration of artificial intelligence and machine learning promises to revolutionize VF analysis by accelerating diagnosis, enhancing pattern recognition, and streamlining disease progression tracking. Moreover, the emergence of virtual reality perimetry and home-based monitoring solutions heralds a future where VF testing becomes more accessible, efficient, and personalized—ultimately improving patient outcomes in the long-term management of retinal diseases.

## Figures and Tables

**Table 1:** Comparison of visual field testing modalities across key parameters and retinal diseases.

Modality	Test Duration *	Area Tested	Patient Burden	Sensitivity/Specificity for Specific Diseases
Confrontation	<1 min per eye	~120–150° (gross field)	Very low	Poor for subtle or early defects; not recommended for screening retinal disease.
Goldmann Kinetic	5–15 min per eye	Central + peripheral (up to 90°)	Moderate	Excellent for mapping peripheral loss in RP and RVO; qualitative only.
Automated Static (HFA)	5–8 min (10-2); 6–12 min (24-2/30-2)	10–30° (protocol-dependent)	Moderate–high	10-2: Good sensitivity (sensitivity 85–90%, specificity 85–95%) for HCQ toxicity screening; central scotoma detection in AMD.
Frequency-Doubling (FDT)	2–3 min per eye	Central ~26° (19 points)	Low–moderate	Potential to detect early neural dysfunction in DR before vascular signs appear. Sensitivity of 90.5% and specificity of 97.6% for sight-threatening retinopathy.
Microperimetry	6–15 min per eye	Central 2–30°	High	Maps paracentral scotomas in AMD (sensitivity 80%, specificity 30.4%); tracks GA progression. 73% sensitivity and 93% specificity for HCQ screening.
Amsler Grid	<1 min (self-test)	Central ~10°	Very low	Identifies metamorphopsia in wet AMD; moderate sensitivity for central defects.

AMD = age-related macular degeneration; DR = diabetic retinopathy; HCQ = hydroxychloroquine; RP = retinitis pigmentosa; RVO = retinal vein occlusion. * Minor variations may occur based on protocol and patient factors.

**Table 2:** Summary of visual field defect patterns and recommended perimetry methods in various retinal diseases.

Disease	Pathophysiology and Overview	Typical Visual Field Defect Pattern	Main Perimetry Method(s)
Age-Related Macular Degeneration (AMD)	Primarily affects adults ≥50 years; central macula destroyed by drusen and/or choroidal neovascularization. Dry (geographic atrophy) and wet (neovascular) forms.	Central scotomas (dark/empty spots) around fixation. In wet AMD: metamorphopsia (“wavy lines”) and large central blind spot. In geographic atrophy: central/paracentral scotomas expand as atrophy grows.	Amsler Grid (self-monitoring) Humphrey 10-2 (central field detail)
Diabetic Retinopathy (DR)/Diabetic Macular Edema (DME)	Microvascular damage from diabetes causes retinal swelling (DME) and ischemia. May involve neuroretinal degeneration before visible vascular signs.	Early: diffuse sensitivity depression (due to neurodegeneration). Later: symmetric nasal/field defects and paracentral scotomas correspond to areas of edema or ischemia.	Frequency Doubling Technology perimetry Blue-on-Yellow perimetry Humphrey 24-2/30-2
Hydroxychloroquine Retinopathy	Long-term hydroxychloroquine use leads to outer retina (photoreceptor with or without RPE) toxicity.	Complete or partial ring scotoma between 2° and 6° from fixation (“bull’s-eye”), often sparing the fovea. In some populations (e.g., Asian patients), pericentral scotoma (≥8° from fixation) can occur.	Humphrey 10-2 (parafoveal retinopathy) Humphrey 24-2/30-2 (pericentral patterns)
Retinitis Pigmentosa (RP)	Inherited rod-cone dystrophy causing progressive photoreceptor and RPE loss. Night blindness (nyctalopia) and progressive peripheral field constriction (“tunnel vision”).	Patchy mid-peripheral defects → coalescing into ring scotoma (~20° from fixation) → constricted tunnel vision. Ring expands peripherally faster than it constricts centrally.	Goldmann kinetic perimetry (especially for peripheral mapping) Humphrey 30-2/60-4 (central support)
Stargardt Disease (Fundus Flavimaculatus)	Autosomal-recessive ABCA4 mutation → lipofuscin accumulation in RPE → macular atrophy. Onset in childhood/adolescence with progressive central vision loss.	Relative or absolute scotoma within central 2–10°. Paracentral islands of preserved sensitivity early on; scotomas enlarge as atrophy progresses. Visual deficits correlate with areas of increased autofluorescence.	Microperimetry (early small-scotoma detection) Humphrey 10-2 (ongoing monitoring)
Central Serous Chorioretinopathy (CSCR)	Serous detachment of neurosensory retina at macula due to choroidal hyperpermeability. Often in males aged 20–50; stress and corticosteroid use are risk factors.	Relative central/paracentral scotoma corresponding to the area of subretinal fluid. Scotoma size/depth fluctuates with fluid resolution or recurrence.	Humphrey 10-2 (central sensitivity) Microperimetry (precise anatomical correlation)
Retinal Vein Occlusion (RVO)	Branch (BRVO) or central (CRVO) retinal vein blockage → inner retina ischemia (capillary nonperfusion) and edema.	BRVO: sectoral (quadrantic) scotomas corresponding to the occluded drainage area (arcuate or altitudinal). CRVO: diffuse central/temporal sensitivity depression	Humphrey 24-2/30-2 (full field mapping) Microperimetry (residual macular scotomas after edema resolves)
Macular Hole	Full-thickness defect in foveal retina, usually from vitreomacular traction. Causes central vision loss and metamorphopsia.	Absolute central scotoma matching the hole’s size.	Humphrey 10-2 (scotoma size corresponds to hole diameter) Microperimetry (post-surgery fixation and residual scotoma)

**Table 3:** Challenges and Limitations of Visual Field Testing in Retinal Diseases.

Category	Challenges/Limitations	Implications/Mitigation
Patient Factors	Subjective test—Relies on attention, comprehension, and cooperation Learning curve: Initial underestimation Unstable fixation in maculopathies Fatigue, arthritis, cognitive/language barriers	Use practice runs; provide clear, language-appropriate instructions Employ microperimetry for unstable fixation Utilize shorter algorithms (SITA-Fast/Faster) and allow breaks
Media Opacities and Coexisting Conditions	Cataracts, vitreous floaters cause diffuse depression or transient scotomas Small pupils and uncorrected refractive errors depress sensitivity Coexisting glaucoma can mimic retinal defects Post-treatment changes (silicone oil or IOP spikes) induces transient field changes	Ensure refractive correction and adequate dilation Correlate with OCT RNFL/GCL maps to distinguish glaucoma vs. retinal defects Schedule testing when post-treatment effects have subsided
Normative Database and Floor/Ceiling Effects	Databases exclude high myopia, comorbidities—misclassification Floor effect: advanced disease registers 0 dB, masking progression Ceiling effect: Early deficits near device limits, hard to detect small changes	Interpret relative to disease context; consider disease-specific norms Use kinetic perimetry or microperimetry when many 0 dB loci Repeat testing to confirm minor threshold shifts
Technical and Environmental Factors	Ambient light and calibration drift affect thresholds Algorithm trade-offs: speed vs. accuracy (SITA-Fast/Faster vs. SITA-Standard) Grid density: 24-2/30-2 may miss small scotomas; 10-2/microperimetry increases test time Inter-device variability limits comparisons	Maintain consistent lighting and calibrate devices regularly Select appropriate algorithm based on patient stamina and precision needs Avoid switching perimeters mid-follow-up; document device parameters
Disease-Specific Issues	HCQ/PPS: 24-2 may miss paracentral scotomas; even 10-2 can miss pericentral PPS defects Stargardt: Eccentric fixation invalidates standard perimetry; microperimetry often needed	Use expanded grids (24-2C, 30-2) or customize test spots Employ microperimetry for eccentric fixation

## Data Availability

Data are contained within the article.
